# Negative play contagion in calves

**DOI:** 10.1038/s41598-020-78748-7

**Published:** 2020-12-10

**Authors:** Verena Größbacher, Alistair B. Lawrence, Christoph Winckler, Marek Špinka

**Affiliations:** 1 Department of Sustainable Agricultural Systems, University of Natural Resources and Life Sciences, 1180 Vienna, Austria; 2 Recipient of a DOC-Fellowship of the Austrian Academy of Sciences, Vienna, Austria; 3 Scotland’s Rural College (SRUC), West Mains Road, Edinburgh, EH9 3RG UK; 4 Roslin Institute, University of Edinburgh, Penicuik, EH25 9RG UK; 5 Department of Ethology and Companion Animal Science, Czech University of Life Sciences, 165 00 Prague, Czechia; 6 Department of Ethology, Institute of Animal Science, 104 01 Prague, Czechia

**Keywords:** Behavioural ecology, Emotion

## Abstract

Play is a strong outwardly directed, emotional behaviour and can contagiously spread between individuals. It has been suggested that high-playing animals could ‘seed’ play in others, spreading positive affective states. Despite the current interest in play contagion there has been no previous attempt to measure the strength of the play contagion effect. The calf (*Bos taurus)* is ideal for testing the strength of play contagion as play in calves is strongly related to energy intake from milk. We manipulated play in calves through their milk allowances and housed the calves in uniform groups all on the same milk allowance (high = UHigh or low = ULow) or in mixed groups with calves in the same group receiving either a high (= MHigh) or low (= MLow) milk allowance. We measured locomotor play using accelerometers on two consecutive days when calves were four and eight weeks old, in order to study play contagion over a protracted developmental window. We anticipated that differences in the level of play contagion between treatment groups would result in difference in the play levels observed in the MLow and ULow individuals. Contrary to our expectations we found that spontaneous play was suppressed in the high-milk calves housed in mixed groups (MHigh), in comparison to calves housed with group mates all receiving high-milk (UHigh). These results are the first to quantify a negative play contagion effect, particularly in a situation of long-term contact, and may suggest that negative contagion has a stronger effect on play behaviour than positive contagion.

## Introduction

‘The rhythm, dance, and spirit of animals at play is incredibly contagious’^1^. This spirit of play radiates from playing animals and can be described as play contagion, the spreading of play behaviour from one animal to another^2–4^. The expressed behaviours of an animal at play may act as stimuli animating play behaviour in other individuals as well as serving as a form of metacommunication, i.e. to signal playful intentions^5^. These play signals may be acoustic (e.g. warble calls in keas^6^, ultrasonic calls in rats^7^, and bark-like vocalizations in piglets^8^) or behavioural (e.g. play-bows in dogs^9^ and play-faces in dogs^9^, and in primates^5^). Dairy calves may signal playful intentions through head-shaking, a behaviour which they perform more frequently when playing with another calf than when playing individually^10^. This aspect of play, when an animal joins a conspecific at play, can be regarded as an expression of behavioural contagion, meaning the behaviour of one animal is stimulated by the similar behaviour of others^11^. However, play contagion is also often discussed in the context of the contagion of positive affective states^2,12,13^ (although see ^14,15^ for opposite arguments). Affective contagion involves one animal perceiving the affective state of another and consequently experiencing a matching affective state^16^. The underlying phenomenon of play contagion is regarded in detail in Held and Špinka^12^. Play contagion would mean that both play behaviour and the positive affective state would be transferred^2^, positively enhancing play motivation as well as the affective state of other animals. This process could be especially relevant in group-housed animals. For example, Held and Špinka^12^ proposed to establish high-playing animals as ‘seeders’ of play behaviour with the aim of improving the wellbeing of other group-mates.

Thus far, a number of studies have demonstrated play contagion. Keas (*Nestor notabilis*) were observed to begin playing upon hearing play-call recordings during a 5-min testing period^6^, while ravens (*Corvus corax*) started playing after seeing a conspecific engage in a short play session with an object^17^. Additionally, highly playful rats (*Rattus norvegicus*) have been demonstrated to stimulate play in their conspecifics^4,18^. However, these studies investigated play contagion only in short-term testing-situations and with reference to specific stimuli. Research regarding the strength of play contagion and how it influences long term behaviour has yet to be conducted.

In the current study we aimed to determine the strength of effect of play contagion over a protracted period of time. Specifically, our objective was to quantify the spreading of spontaneous play in the home-pen of animals, not in a testing-situation. For this purpose, we recorded play behaviour of dairy calves, a social species with a high motivation to perform play and for which group-housing is strongly recommended^19,20^. Ungulates primarily express locomotor play, a form of play consisting of vigorous and energetic movements with elements of running, jumping and bucking^19,21^. Locomotor play is often performed in parallel with other animals and can then be considered social locomotor play^21,22^. Studies have shown that locomotor play in calves is reduced by space restrictions^19^, painful procedures^23^ as well as low milk allowance and concomitant low energy intake^24,25^. Based on the relation of locomotor play and energy intake, we induced high and low play levels in calves with high and low milk allowances. We then compared play of calves in uniform groups (UHigh and ULow), where all calves received the same amount of milk, to play of calves in mixed groups, with calves on a high-milk-diet (MHigh) cohabiting with calves on a low-milk-diet (MLow). With this experimental setup we aimed to address the following questions: 1. How strong is the effect of play contagion on spontaneous locomotor play of dairy calves? 2. What are the effects of different milk allowances and play contagion on the synchrony of play? 3. What are the effects of other factors such as health, ambient temperature and time of day on the incidence of locomotor play? We hypothesized that: 1. Through the play contagion effect, low-milk calves in the mixed group (MLow) will perform more play than calves in the uniform low-milk groups (ULow); 2. Calves playing more as a result of the contagion effect (MLow) and through a higher milk allowance (MHigh and UHigh) will have higher levels of dyadic play, i.e. calves playing synchronously; 3. Play is reduced by other factors such as impaired health and ambient temperature.

## Animals, material and methods

### Ethical considerations

This study was carried out in accordance with the guidelines for ethical treatment of animals of the International Society of Applied Ethology. It was approved by the Institutional Animal Care and Use Committee of the Institute of Animal Science and the Czech Central Committee for Protection of Animals, Ministry of Agriculture (Permit Number 27356/2016-MZE-17214). Calves on the low-milk schedule received a milk allowance of approx. 12% of their body weight, equalling the traditional calf feeding practices^26^.

### Animals and housing

The study was conducted at the Netluky research station of the Institute of Animal Science in Prague, Czech Republic. Data were collected from August 2016 until April 2017.

Seventy-two Holstein Friesian dairy calves (31 heifers and 41 bulls) were included in the study. Calves were separated from their dams at approximately 12 h after birth and housed individually either in outdoor hutches or in individual pens in a naturally ventilated open barn equipped with curtains. In both cases, the area available for each calf was 1.4 m × 1.4 m straw-bedded lying area and 1.2 m × 1.2 m solid walking area. While in individual housing, calves were fed 3 l of milk twice per day through teat-buckets at 06.00 and 18.00 and received concentrates and water ad libitum. Calves entered the experiment at an average age of 13.3 ± 3.1 days (mean ± S.D.) and were then housed in groups of three. Calves were allocated to groups balanced by sex, age and weight. Groups entered the experiment consecutively with 1–2 groups per week. Groups were housed in a naturally ventilated open barn with curtains. Group pens were 10.1 m^2^ consisting of a straw-bedded lying area (4.2 m × 1.4 m; approx. 2.0 m^2^ per calf) and a concrete walking and feeding area (3.5 m × 1.2 m). The group pens were covered with visual barriers in order to avoid direct visual contact of other calves. The visual barriers in front of the respective pens were removed in order to allow video recording; however, groups that were recorded simultaneously were allocated in the barn in a way that precluded visual contact without the front visual barriers of the pens. The calves received water, hay and concentrates ad libitum, offered in buckets and were provided with fresh straw bedding three times per week. All routine farm work was done before 10.00. Calves were hot-iron disbudded at 24.4 ± 3.1 days of age (mean ± S.D.). Disbudding wounds are painful for more than three weeks^27^, however no difference in play behaviour after disbudding was found after 27 hours^23^, thus we do not expect an effect of disbudding on play in our study. On the recording days, the air temperature in the barn ranged between -4.5 °C and 29 °C with the average (± S.D.) being 7.9 (± 8.6) °C.

### Experimental design and procedures

#### Experimental design

**Figure 1:**
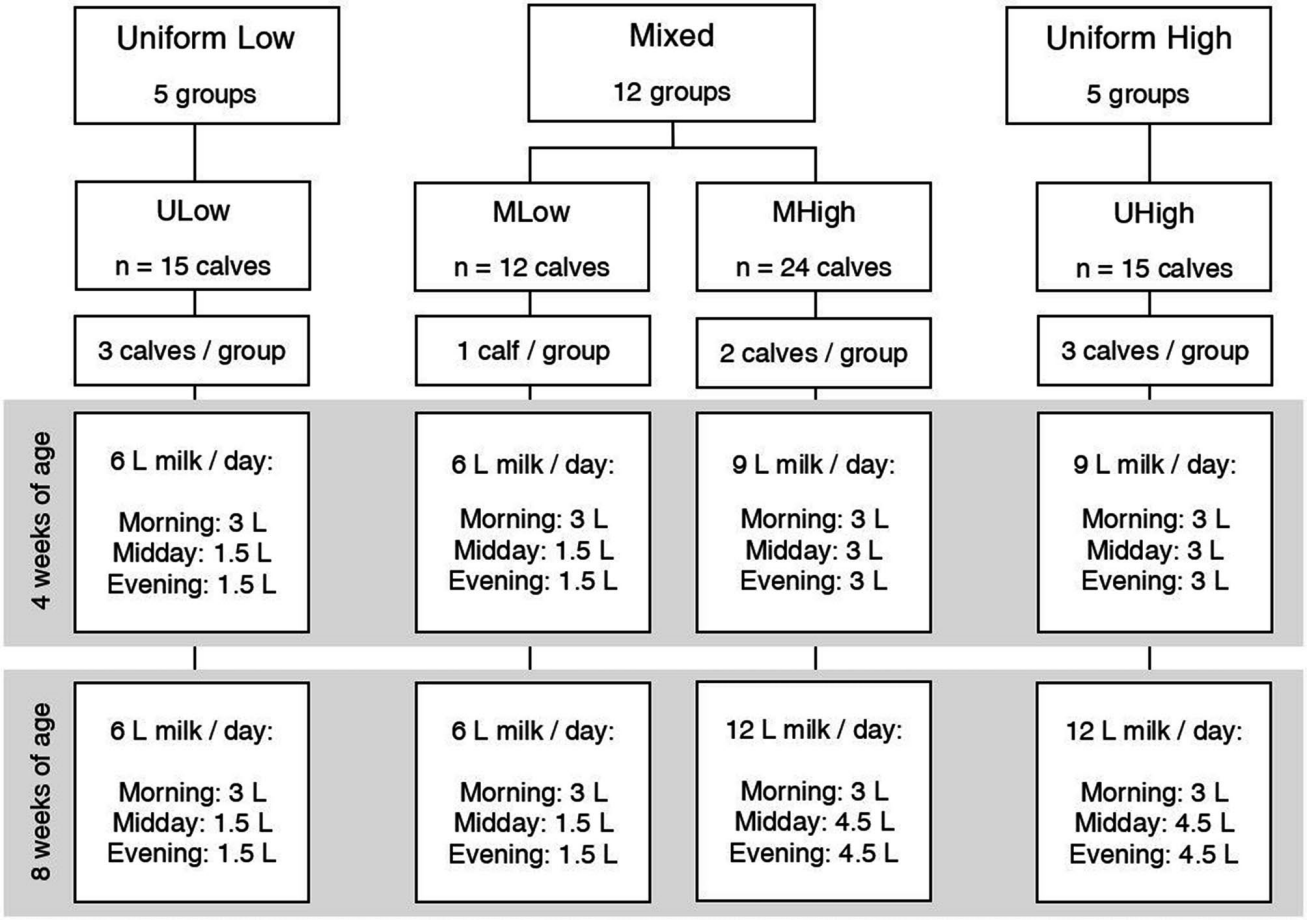
Experimental design of treatments, group composition and milk allowance. Sample size is the number of calves included in statistical analysis.

Groups were allocated to treatments balanced by sex composition, age, weight and point of time entering the experiment. Milk allowance, group composition and number of groups assigned to each of the treatments are displayed in Fig. 1. Calves in all treatments received three milk meals per day at approximately 06.00, 12.00 and 18.00. All calves were offered 6 l of milk per day at the beginning of week 3. For UHigh and MHigh calves, the offered milk was gradually increased to 9 l of milk per day in week four and 12 l of milk per day in week six (Fig. 1). Therefore, the total milk amount offered from the start of the experiment until the end of week eight (42 days) was 240 l for ULow and MLow calves and 420 l for UHigh and MHigh calves. Milk was offered in teat buckets. Calves were tethered for the duration of the milk meal using neck collars and were released when all calves of the group had finished their meals (i.e. calves had either emptied the buckets or stopped drinking milk; approx. 5 min). If calves did not finish the offered milk meal, the volume of the remaining milk amount was measured. The volume of unconsumed milk was then summed from the point of entering the experiment until the respective day of behaviour recording. For ULow and MLow the total volume of unconsumed milk amounted to 0.3 ± 0.9 l (mean ± S.D.; median/interquartile range: 0/0 – 0). For UHigh and MHigh the total volume of unconsumed milk amounted to 16.7 ± 18.0 l (median/interquartile range: 10/2.5–25). The average daily amount of milk refusal was 0.1 ± 0.3 l and 0.3 ± 0.6 l, when calves were four weeks and eight weeks old, respectively.

Data from two groups were excluded from statistical analysis: in one ULow group, a calf died from health issues unrelated to the experiment and one UHigh group was treated for severe diarrhoea for a prolonged time and therefore was not offered 12 l of milk in order to avoid further digestive problems.

#### Health and weight assessment

Calves’ health state was assessed once per week by two assessors. The following indicators of compromised health were recorded: diarrhoea, coughing/sneezing and increased respiratory rate (adapted from Gratzer, et al.^28^; Supplementary Table 1). The overall health score was set to 0 when calves showed no or one symptom of diarrhoea or coughing/sneezing and 1 when calves showed either combined diarrhoea and coughing/sneezing or increased respiratory rate. The ratio of calves with a health score of 1 is shown in Table 1.

**Table 1:** Ratio of calves with a health score of 1 by treatment and age group.

	Treatment			
	ULow	MLow	MHigh	UHigh
Four weeks of age	8/15	4/12	6/24	8/15
Eight weeks of age	6/15	3/12	12/24	6/15

Calves were weighed once per week between Monday and Thursday. To allow for comparison, daily weight gain for every respective week was calculated and weights were subsequently corrected for Monday as reference weighing day. Body weights from the start until the end of the experiment (three to eight weeks of age) are presented in Supplementary Figure S1. These data show that higher milk provision in MHigh and UHigh calves resulted in faster growth.

### Quantification of play behaviour

#### Data recording

Locomotor play behaviour of calves was quantified through leg-attached accelerometers, using a previously validated method^29^. In this study, accelerometers were used to record running, turning and bucking/buck-kicking, as defined in Größbacher, et al.^30^. The data used to validate accelerometer recordings for these behaviours^30^ were a subset of the data used in this study. Accelerometers (HOBO Pendant G Acceleration Data Logger, Onset Computer Corporation, Pocasset, MA, USA; product specifications described in detail in Luu, et al.^29^) were attached to calves’ hind legs with elastic cohesive bandages. The accelerometers were oriented with the x-axis perpendicular to the ground. Acceleration was measured on the vertical axis at 1 Hz, i.e. with one measurement per second, from 05.00 until 23.04 on two consecutive days (Tuesday and Wednesday) when calves were four and eight weeks of age and recordings were stored on the device. Accelerometers were fitted to calves from the evening before until the morning after recording days, after being programmed with an optical infrared base station with USB interface and the HOBOware Pro Software (Version 3.7.8; Onset Computer Corporation, Pocasset, MA, USA).

#### Behaviour classification

Data processing was performed in SAS 9.4. Always 10 acceleration measurements, representing a period of 10 s each, were evaluated. These 10 s periods were categorized into lying, standing or play behaviour using quadratic discriminant analysis. This categorization was based on six predictor variables, which were calculated for each period with the respective 10 values: mean of two highest acceleration measurements, mean of two lowest acceleration measurements, variance, maximum of absolute value of change in acceleration measurement, mean change in acceleration measurements, and total sum of absolute values of change in acceleration measurements^30^.

In order to develop the discriminant function, a reference data set was created with randomly selected short sections of accelerometer data obtained from recordings of calves in this study. This reference data set consisted of 52 recordings with a mean (± S.D.) duration of 37.8 ± 16.8 min. Lying, standing and play behaviour were visually identified from video of these recordings applying one-zero-sampling of the respective 10 s periods, for which predictor variables were calculated. This was used as the gold standard.

The discriminant function was then applied to the entire data set in two steps to identify periods that contained locomotor play, i.e. included events of running, turning and/or bucking^30^, based on the six predictor variables: The first discriminant function classified the acceleration data into lying and standing, based on equal prior probabilities (50:50 chance of both behaviours occurring). The second discriminant function classified all standing-periods according to their presence or absence of locomotor play, based on prior probabilities of the reference data set (3:97 chance of play occurring across all treatments). The transitions from lying to standing and vice versa were almost always falsely classified as playing, as identified from video, and reclassified into standing.

The validation of processing the raw acceleration data was accomplished through checking the agreement between the acceleration-based method and visually identified play of the reference data set^30^. It proved that although the absolute play-levels were overestimated with the acceleration method, the method was able to truthfully quantify the inter-individual differences in locomotor play in dairy calves^30^.

### Data analysis

#### Data processing

The last four minutes of each recording were omitted to obtain observation durations of exactly 18 h. The number of 10 s periods of locomotor play was converted into minutes of locomotor play per recording day (18 h). Recordings were excluded for the duration of disturbance when any calf in the barn escaped their pen or a person entered the pen. If more than 1 h was missing or compromised, the entire recording day was excluded for the calves affected. If less than 1 h of the recording was missing, locomotor play was calculated on a per hour basis and extrapolated to the ‘standard’ duration of 18 h. Out of 264 recordings (4 recordings per calf overall with 2 recordings at the age of four and eight weeks, respectively), 17 recordings were excluded or missing and in 13 recordings a mean (± S.D.) of 23.9 ± 13.0 min were missing and locomotor play duration and bout frequency were extrapolated. This resulted in 245 recordings, i.e. data points, included in the model. Play bouts were assessed by counting standalone periods of play, i.e. single 10 s periods not proceeded and not followed by periods classified as play were counted as one play bout, or by counting consecutive periods of play, i.e. two or more play periods occurred in a row as one play bout. Mean bout durations were assessed by recording the duration of each bout, e.g. a play bout consisting of one play period was recorded as 10 s and a play bout consisting of 3 play periods was recorded as 30 s.

Individual play was defined as one calf performing play in a 10-s-period when no other calf in the group was performing play. Dyadic play was defined as one calf performing play in the same 10-s-period as any one other calf of the group. Individual and dyadic play were calculated as minutes per recording day (18 h). Data were only included when observations of all three calves of the group were available. If data of one of the calves in the group were partially missing, observations of the other calves for the same period of time were excluded. Then the duration of individual and dyadic play was extrapolated on a per hour basis as described above. 237 recordings, i.e. data points, were included in the analysis, whereof 15 recordings were extrapolated.

The observed and randomly expected proportion of dyadic synchronized play were calculated on the basis of dyads, i.e. the combination of always two calves of a group, according to Šilerová, et al.^31^. Both were calculated for each pair combination:

$$ Sync_{obs} = \frac{{\left( {2*C_{sync} } \right)}}{{\left( {C_{A} + C_{B} } \right)}} $$

$$ Sync_{exp} = \frac{{(2* C_{A} *C_{B} *1/P_{dyad} )}}{{\left( {C_{A} + C_{B} } \right)}} $$

where *C*_*sync*_ is the number of synchronous play periods of the pair, *C*_*A*_ is the total number of play periods of calf A, *C*_*B*_ is the total number of play periods of calf B and *P*_*dyad*_ is the total number of recorded periods for each dyad. The randomly expected proportion of play is the proportion of synchronized play occurring by chance if the calves played independently of each other^31^.

Triadic play was defined as all three calves of a group performing play in the same 10-s-period and calculated as minutes per recording day. Only periods in which data for all calves of the group were available were included. Extrapolation of play duration (due to partially missing data) was done in 10 out of 79 recordings.

### Statistical analysis

All data was analysed in SAS Version 9.4. Five separate linear mixed effects models were run with total duration of play, frequency of play bouts, mean bout duration, duration of dyadic play or duration of individual play as dependent variables. Treatment (ULow, MLow, MHigh, UHigh), age (week four, week eight) and overall health score (0,1) were included as fixed class effects, while volume of unconsumed milk and maximum daily temperature were included as fixed quantitative effects, i.e. as covariates. Age (week), nested in calf and group were included as random effects. Furthermore, the date of recording was included as a crossed random effect. The same model was used for all dependent variables. Initially, the full model contained the interaction effect of treatment and age, however this was never significant and therefore removed from the model. An auto-regressive covariance structure was selected based on the Akaike Information Criterion (AIC). All models were visually inspected for normal distribution of residuals devoid of skewness.

## Results

### Play duration and play structure

**Figure 2:**
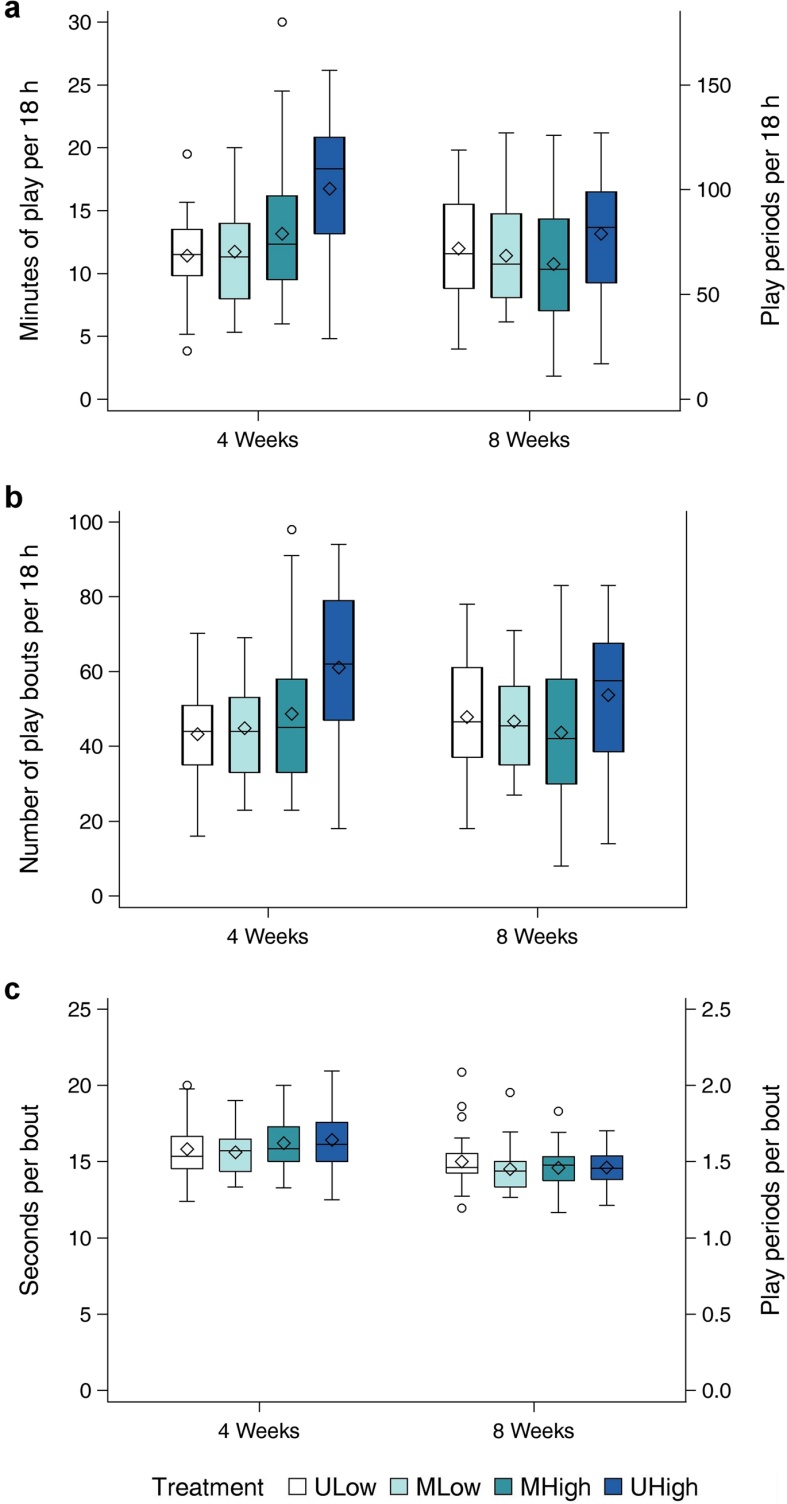
Total duration of locomotor play (**a**), frequency of locomotor play bouts (**b**) and mean duration of locomotor play bouts (**c**) by treatment and age. Diamonds represent arithmetic means.

There was a significant effect of treatment on both total play duration and on the frequency of play bouts (Fig. 2, Table 2) with UHigh calves showing higher play levels with a higher total duration and more numerous bouts of locomotor play than calves in the other three treatments (pairwise comparisons: all *p* ≤ 0.01). Both MLow calves and MHigh calves played at a similarly low level as ULow calves in terms of total duration and bout frequency (pairwise comparisons: all *p* > 0.1). Mean duration of play bouts did not differ between treatments. Age did not affect total duration and bout frequency of play, but mean duration of play bouts decreased from week four to week eight.

**Table 2:** Estimated least square means ± standard error (SE), *F* and *p*-values for treatments and age (weeks) from statistical analysis for all measured variables. Means within each row with different superscript letters differ at *p* < 0.05 in pairwise comparisons.

Number of calves	Treatment				Treatment effects		Age		Week effects	
	Uniform low (ULow) n = 15	Mixed low (MLow) n = 12	Mixed high (MHigh) n = 24	Uniform high (UHigh) n = 15	F-Value	*p*-Value	Week four	Week eight	F value	*p *Value
**Play total duration**										
(min/18 h)	11.4 ± 0.9^a^	10.9 ± 0.9^a^	12.3 ± 0.7^a^	15.2 ± 0.9^b^	5.08^1^	0.003	12.9 ± 0.6	11.9 ± 0.6	2.32^3^	0.131
**Play bout frequency**										
(bouts/ 18 h)	44.1 ± 3.3^a^	43.1 ± 3.1^a^	47.3 ± 2.5^a^	58.4 ± 3.3^b^	4.79^1^	0.004	47.9 ± 2.1	48.5 ± 2.0	0.08^3^	0.778
Play mean bout duration										
(sec/bout)	15.5 ± 0.4	15.0 ± 0.3	15.4 ± 0.3	15.3 ± 0.4	0.88^1^	0.454	16.0 ± 0.2^a^	14.6 ± 0.2^b^	39.60^3^	< 0.001
**Individual play duration**										
(min/18 h)	8.4 ± 0.7^a^	8.3 ± 0.7^a^	9.6 ± 0.6^a^	11.6 ± 0.7^b^	4.60^2^	0.005	9.3 ± 0.4	9.7 ± 0.5	0.35^3^	0.554
**Dyadic play duration**										
(min/18 h)	2.3 ± 0.2^a^	2.0 ± 0.2^a^	2.4 ± 0.2^ab^	2.7 ± 0.2^b^	3.19^2^	0.028	2.7 ± 0.2^a^	2.0 ± 0.2^b^	5.11^3^	0.026

^1^F_3,89_.

^2^F_3,85_.

^3^F_1,97_.

Calves displaying signs of health impairments had a lower total duration (F_1,89_ = 9.60, *p* = 0.003) and bout frequency (F_1,89_ = 9.16, *p* = 0.003) of play. Moreover, total duration and bout frequency decreased with higher volumes of unconsumed milk (total duration: F_1,89_ = 8.97, *p* = 0.004; bout frequency: F_1,89_ = 9.62, *p* = 0.003). Higher maximum daily temperature decreased the total duration (F_1,89_ = 5.35, *p* = 0.023) but did not affect the bout frequency (F_1,89_ = 1.85, *p* = 0.177) of play. Mean bout duration was not affected by health impairments (F_1,89_ = 0.25, *p* = 0.621) and volume of unconsumed milk (F_1,89_ = 0.80, *p* = 0.375) but decreased with a higher maximum daily temperature (F_1,89_ = 10.93, *p* = 0.001).

### Synchrony of play

**Figure 3:**
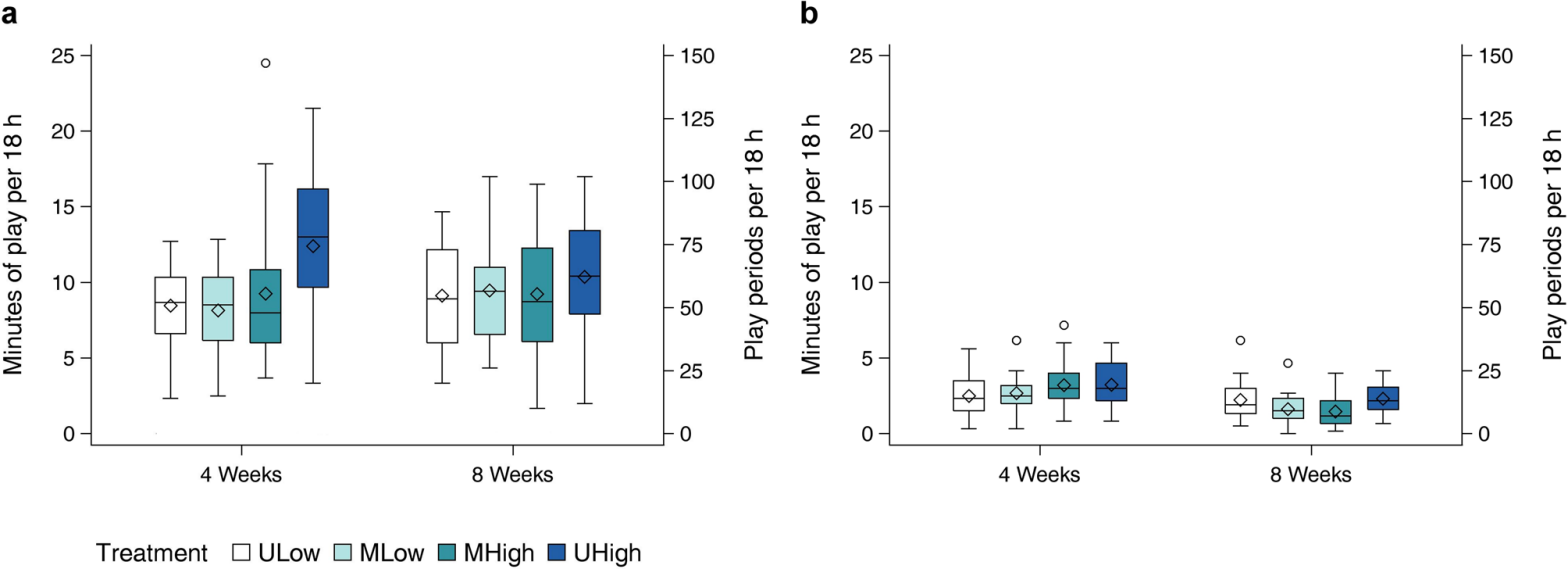
Duration of individual play (**a**) and dyadic play (**b**) by treatment and age. Diamonds represent arithmetic means.

Individual play, i.e. play of only one calf at any time, accounted for the majority of play duration with an average of 9.5 ± 3.8 min per 18 h. UHigh calves performed more individual play than calves of all other treatments (ULow: F_1,85_ = 10.11, *p* = 0.002; MLow: F_1,85_ = 10.86, *p* = 0.001; MHigh: F_1,85_ = 5.21, *p* = 0.025; Fig. 3, Table 2). Neither the other three treatments nor the two age groups differed from each other in respect to individual play (all *p* > 0.1). Dyadic play, i.e. simultaneous play observed by any pair of calves in a triad, constituted 8.8 ± 5.8% of the total play, and was approximately eight times higher than randomly expected by chance at 1.1 ± 0.4% (Supplementary Figure S2). It was performed for on average 2.4 ± 1.4 min per 18 h and differed by treatment (Fig. 3, Table 2) with MLow calves showing less dyadic play than UHigh calves (F_1,85_ = 8.56, *p* = 0.004). MLow calves also tended to perform less dyadic play than MHigh calves (F_1,85_ = 3.77, *p* = 0.056) but they did not differ from ULow calves (F_1,85_ = 1.23, *p* = 0.271). ULow calves performed less dyadic play than UHigh (F_1,85_ = 4.25, *p* = 0.042) calves but did not differ from MHigh calves (F_1,85_ = 0.23, *p* = 0.635). UHigh and MHigh calves performed dyadic play for the longest durations and did not differ from each other (F_1,85_ = 2.54, *p* = 0.115). Moreover, dyadic play decreased with age (Table 2).

Dyadic play decreased with increasing maximum daily temperature (F_1,85_ = 7.74, *p* = 0.007) but individual play was not affected by it (F_1,85_ = 2.25, *p* = 0.137). Both decreased with impaired health (Individual: F_1,85_ = 4.20, *p* = 0.044; Dyad: F_1,85_ = 12.92, *p* < 0.001) and an increasing volume of unconsumed milk (Individual: F_1,85_ = 8.65, *p* = 0.004; Dyad: F_1,85_ = 4.05, *p* = 0.047).

Triadic play, i.e. all three calves in a group playing simultaneously, was performed for a total duration of 0.6 ± 0.5 min (mean ± S.D.) in the ULow groups, for 0.6 ± 0.6 min in the MLow-MHigh groups and for 0.9 ± 1.2 min in the UHigh groups. Statistical testing was not performed for triadic play because the duration of this category of play was by definition the same for MLow and MHigh calves, thus a statistical model differentiating the four treatment levels could not be developed.

### Temporal distribution of play

**Figure 4:**
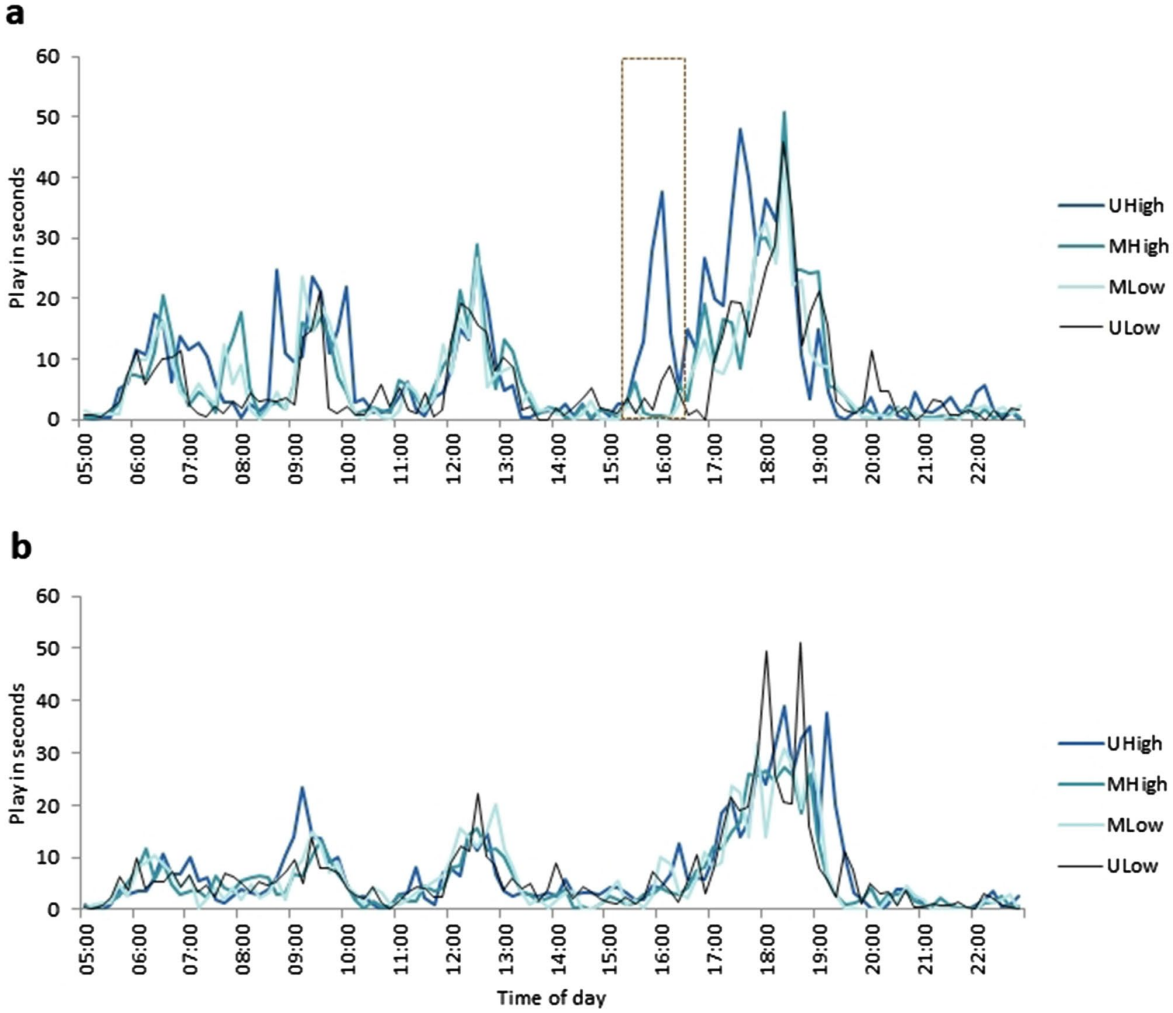
Distribution of locomotor play across the day for four week (**a**) and eight week (**b**) old calves. Data were calculated as seconds of locomotor play per ten minutes of observation.

Calves performed locomotor play in a highly synchronous temporal distribution across treatments and throughout the day (Fig. 4). In all treatments play peaked around the times of milk feeding (06:00, 12:00, 18:00) and other husbandry work (09:00–10:00), with the highest incidence of play in the evening. Calves of the UHigh treatment showed a higher persistency of peaks and an additional play peak in the afternoon when four weeks old.

## Discussion

In this study we aimed to estimate the strength of effect of play contagion, i.e. the potential of transmitting play behaviour from one calf to another. We recorded the locomotor play of calves in mixed (M) groups with one calf on a 6 l milk allowance (MLow) being grouped with two calves each on a 12 l milk allowance (MHigh). We compared M group play levels to uniform groups in which all three calves had a milk allowance of either 6 l (ULow) or 12 l (UHigh). We found increased play only when all calves of a group were on a high milk allowance (UHigh). Despite receiving the high milk allowance, play of MHigh calves was as low as that of MLow and ULow calves. This play depression in MHigh calves indicates a negative contagion effect.

In this study, we effectively manipulated calf locomotor play with different milk allowances in uniform groups, replicating the increase in spontaneous locomotor play in calves fed higher amounts of milk (9 l per day^32^ or 12 l per day^25^) in comparison with lower amounts of milk (5 l per day^32^ or 6 l per day^25^) reported in previous studies. Both previous studies linked the increase in play with higher digestible energy (DE) intake, a relationship that is substantiated with a positive correlation of both, DE intake and weight gain, with running, a measure of calf locomotor play^33^. Indeed, play consumes a certain amount of energy in ungulates, e.g. play is estimated to require 2% of energy intake in pronghorn fawns^34^ and 0.9% in white-tailed deer^35^. Although these proportions may appear low, reducing the milk provision by one third in white-tailed deer reduced play proportionally and strongly increased grazing; nevertheless the deer continued to play^35^. This persistence of play in the context of overall reductions in energy intake indicates the importance of play but also that the motivation to play is affected by factors other than the physical development.

Alternatively to play increasing with a higher DE intake, the increase of play with a higher milk allowance may be explained by a connection between a high milk intake and positive affective states. In the current study, the diurnal pattern of play peaking at milk feeding times may indicate more immediate effects of milk consumption on locomotor play other than overall DE intake or weight gain. Drinking of milk may relate to an affectively valenced context, which is substantiated by the strong motivation of pre-weaned ruminants to obtain milk (see e.g.^36–38^). Indeed, detecting and obtaining a food reward may stimulate a positively valenced affective state of high arousal^39^. We hypothesize that milk can elicit such an affective response and that in the context of milk feeding, calves may express positive and highly aroused affective states as locomotor play behaviour. Our data do not allow us to conclude whether calves played in anticipation before the milk meal or after it in consequence of ‘obtaining the reward’, however there are arguments supporting both possibilities. In our study, milk was administered on a fixed schedule, which would allow calves to anticipate these events. Overall, animals are reported to increase their activity in anticipation of a reward^40^. While activity and play are not to be equated, high activity may be associated with play and thus may explain the diurnal multimodal pattern with peaks of play at times of milk meals and other husbandry work. Similarly, anticipation of a reward eliciting play may be seen in rats playing when anticipating tickling by a human hand, a model mimicking rat social play^13^ and in pigs playing when anticipating rooting material and chocolate raisins^41^. However, in pigs overall play increased when receiving an expected reward^42^. Concurrently, milk consumption may have raised play levels through the reward associated with satisfaction of feeding motivation. This effect was observed in meerkats, which doubled the duration of playing and reduced food begging for one hour after supplemental feeding^43^. Regarding calves on a 6 l milk allowance, their lowered play levels may be interpreted as consequence of both, frustration and hunger, when their milk meal ceased before obtaining full satiety. Notwithstanding, our results support these explanations only in uniform groups with all calves receiving the same milk allowance. Other possible explanations for the performance of play around feeding, such as strengthening social bonds and/or preventing food-motivated aggression/competition^44,45^, need to be also examined in further studies.

In mixed groups, containing calves with different milk allowances, locomotor play did not generally increase with milk quantity. Despite receiving up to 12 l milk per day, MHigh calves were lower in their total play duration, play bout frequency and individual play than UHigh, and played at the same level as ULow. Correspondingly, there was no evidence that play of MLow calves increased through a hypothesized play contagion effect. This outcome emphasizes the strong influence of the social environment on play behaviour. Likewise, in pigs a strong group effect on play behaviour has been observed with the litter affecting changes in locomotor play before and after weaning^46^. Here, contagion was rendered secondary to other factors as Brown, et al.^46^ reason that contagion can only explain litter differences when it accounts for reduced play through negative contagion, a possible explanation that we suggest for our findings.

The results of our study raise the question whether the depression of play in MHigh calves was caused by the absence of another (third) calf receiving 12 l of milk or by the presence of a calf receiving 6 l of milk. Our results do not substantiate the argument that MHigh calves showed reduced locomotor play because a third and equally motivated play partner was missing, i.e., because the amount of mutual play stimulation was reduced. If this were the case, then the effect should have been particularly strong on the dyadic play. However, this was not the case in our data: dyadic play of MHigh calves was not lower than of UHigh calves, while it was the individual play that was clearly reduced in MHigh calves, compared to UHigh calves. Furthermore, the argument that play was reduced because of a missing third play partner could be related to a more general idea that play decreases with decreasing group size. We are not aware of any study supporting this idea, while a study in meerkats observed no relationship between group size and play levels^43^.

Alternatively, play of MHigh calves may have been reduced by the presence of a MLow calf. The MLow calf could be argued to be in a negatively valenced affective state due to the lower milk provision and may have altered the affective state of its group-mates. In contrast, UHigh calves may have had increased play levels stimulated by a prevailing positive affective state in the absence of a negatively valenced emitter. The diurnal pattern displaying play levels across the day may serve as indicator of this with an additional peak in the afternoon in UHigh calves, a point in time for which we cannot provide an explanation of external stimulation. Both positive and negative emotional / affectively valenced contagion can occur in groups of animals^3,47^. In our study, contagion of the negative affect from MLow calves may have superseded possible contagion of positive affective states from MHigh calves. Across various taxa, a phenomenon of negativity bias has been observed, consisting of animals paying more attention to negative situations and stimuli than to their positive equivalents^48^. For instance, ravens showed a pessimism-bias after observing a conspecific experiencing a negative affective state but did not show an optimism-bias after observing a conspecific with positive affect^49^. Similarly, the presumed expression of a negative affective state (e.g. resulting from hunger) in MLow calves may have received more attention than the expression of a positive affective state due to e.g. satiety in the MHigh calves. Nevertheless, negativity bias is not all prevailing. In a balanced study on domestic pigs, contagion of both negative and positive affects was documented^3^. Further studies are needed to find out under what conditions and to what extent calves in a more negative affective state influence the affective states^20^ and/or the expression of natural behaviours in their group mates and thus when positive affective contagion may arise and when it is repressed by negative contagion.

In addition to milk and contagion effects, play behaviour of calves was affected by age. In previous studies, the relationship of play and energy intake was either weaker in older calves when weaned^33^ or it was only found at younger ages (two^32^ and three^25^ weeks old). In older calves the duration of locomotor play either decreased after reducing the milk allowance (six weeks old)^32^ or increased when calves compensated a low milk allowance with solid food, therewith increasing their DE intake (five weeks old)^25^. By comparison, calves in our study only had a lower mean bout duration and less dyadic play when eight weeks old compared to four weeks old. We reason that in our study reduced play in older calves was not affected by energy intake through milk, but that the lower relative space allowance reduced these play measures^19^. Especially dyadic play may have been affected by lack of space when calves were older and larger, dissuading each other when playing at the same time. Calves may have also shifted from locomotor to social play when eight weeks old, as an increase of social play with age has been reported previously in calves^22^. Although the accelerometers will also have identified components of social play concomitant with vigorous movements, we have not separately recorded elements of social play and therefore cannot draw firm conclusions on this.

Play behaviour of calves was also sensitive to other influences such as health impairments, reduced milk intake and high ambient temperature. Both, volume of unconsumed milk and impaired health, i.e. symptoms of diarrhoea, coughing/sneezing and increased respiratory rate, reduced the duration of total play, individual play and dyadic play as well as play bout frequency but had no effect on mean bout duration. Thus, calves with health impairments and reduced milk intake were less likely to start playing, but when they played the duration of play bouts was unaltered. To our knowledge, this is the first indication that the health state can affect an individuals’ play behaviour. Bertelsen and Jensen ^10^ previously found a linkage between reduced play and mildly impaired health of the companion calf but contrary to our study, they did not find effects of the focal calves’ own health on their play. It should be emphasized however, that none of their calves were clinically ill or showed obvious sickness behaviour^10^. Our results suggest that play may act as a sensitive indicator for animal health^12^. It may gradually decrease with the degree of impaired health but not completely diminish unless animals are affected by severe clinical illness. However, further studies are needed to support the play-health-relationship. In the case of higher ambient temperature, total duration, mean bout duration and dyadic play were reduced and thus play may also serve as a useful indicator for heat stress. It has to be considered though, that the expression of play is highly variable between individuals and influenced by a multitude of factors that we may not have controlled for, thus part of the variation cannot not be explained even by extensive models. However, overall, our findings substantiate that higher levels of play may indicate the absence of negative welfare^12,14^.

In conclusion, our study suggests the possibility of long-term negative play contagion, i.e. the suppression of play behaviour by conspecifics. As it is the first study indicating such effect, confirmation by further research is needed. Specifically, we have shown that in small groups of calves, individuals that are less well fed and therefore presumably in a more negative affective state may reduce locomotor play behaviour of their well-fed group-mates. More generally, our study documents that locomotor play may be more strongly affected by social environment than by energy intake.

## Supplementary Information

Supplementary Information

## Data Availability

The datasets generated and analysed during the study are available in the Figshare Data repository.
