# Supplementary material for: Negative play contagion in calves
Source: Sci Rep. 2020 Dec 10;10:21699. doi: 10.1038/s41598-020-78748-7 (PMC7728765; doi:10.1038/s41598-020-78748-7)
Supplement: Supplementary file 1 — Supplementary Information [file 41598_2020_78748_MOESM1_ESM.pdf]

Negative play contagion in calves

Verena Größbacher[1,2*], Alistair B. Lawrence[3,4], Christoph Winckler[1] & Marek Špinka[5,6]

[1] Department of Sustainable Agricultural Systems, University of Natural Resources and Life Sciences, 1180 Vienna, Austria
[2] Recipient of a DOC-Fellowship of the Austrian Academy of Sciences
[3] Scotland's Rural College (SRUC), West Mains Road, Edinburgh EH9 3RG, UK
[4] Roslin Institute, University of Edinburgh, Penicuik, EH25 9RG, UK
[5] Department of Ethology and Companion Animal Science, Czech University of Life Sciences, 165 00 Prague, Czechia
[6] Department of Ethology, Institute of Animal Science, 104 01 Prague, Czechia
*Corresponding author: verena.groessbacher@boku.ac.at

Supplementary Material

Supplementary Table 1. Health assessment scheme for diarrhoea, coughing/sneezing and respiratory rate. Score 0 = no signs of illness; Score 1 = mild, long-term action necessary; Score 2 = severe, immediate action necessary.

Criterion	Assessment
Diarrhoea<br>Faeces are loose and watery, strong smell, abundant, calf seems low, weak, doesn't want to stand, ears are hanging.	Assessment of the tail, deposition of wet or dry faeces around the tail, observing of defecation<br><br>0 = no symptoms or one symptom e.g. colour, odour, amount or consistency altered<br><br>1 = multiple symptoms
Coughing/Sneezing<br>The calf is coughing or sneezing.	0 = no symptoms or calf is coughing or sneezing 1-2 times, rather dry, no other symptoms<br><br>1 = calf is coughing or sneezing more than two times
Respiratory rate<br>Normal respiratory rate for a calf is 25-35 breaths per minute.	Assessment from angular to the right behind the calf. Observation of thorax, costal arch, abdominal wall and motion of flanks of thorax. Counting the intake of breath for 30 seconds and multiply by two<br><br>0 = no symptoms or normal high values that could be explained by context (e.g. agitation, recent stress,…)<br><br>1 = increased respiratory rate, abdominal or oral respiration, coastal arch expanded

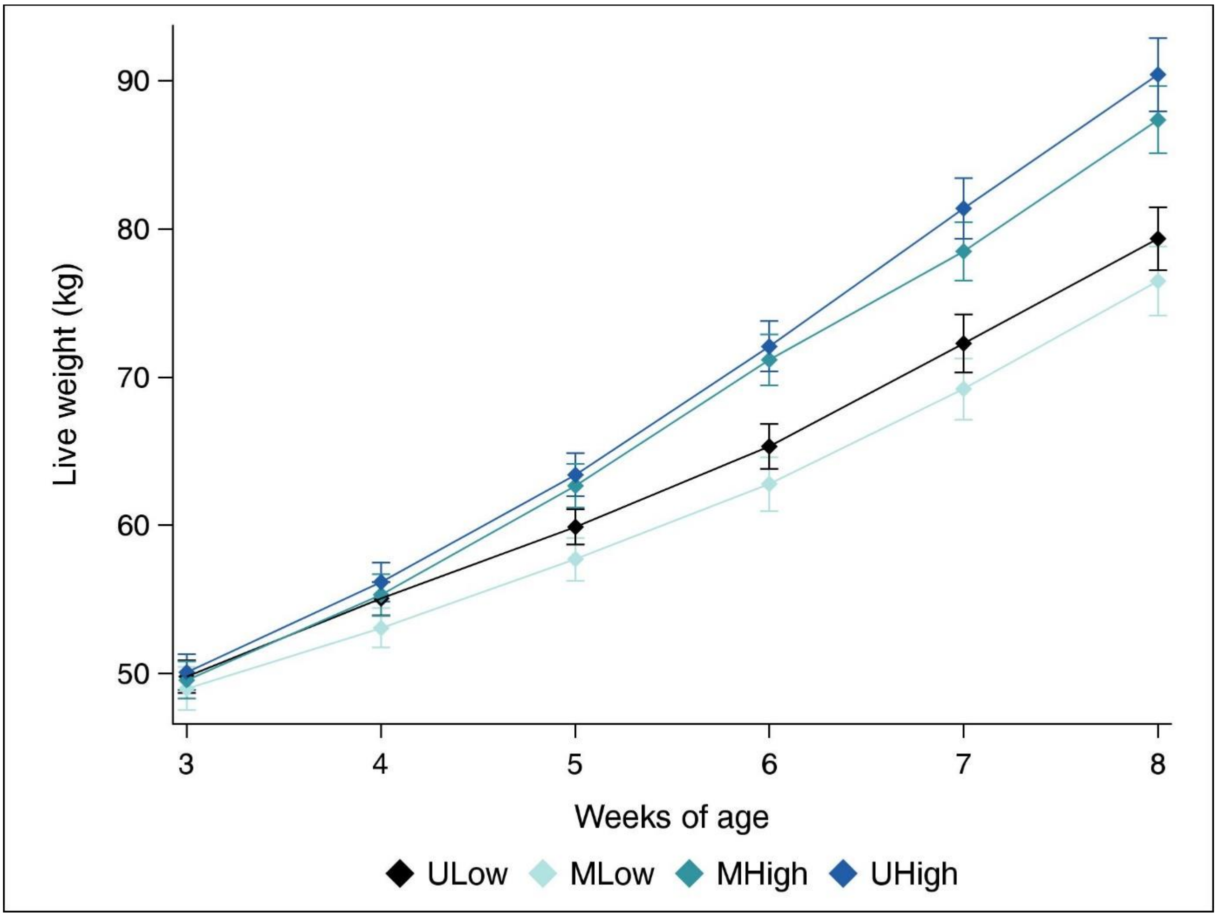

Supplementary Figure S1. Arithmetic means and standard errors of live weight per treatment and across weeks

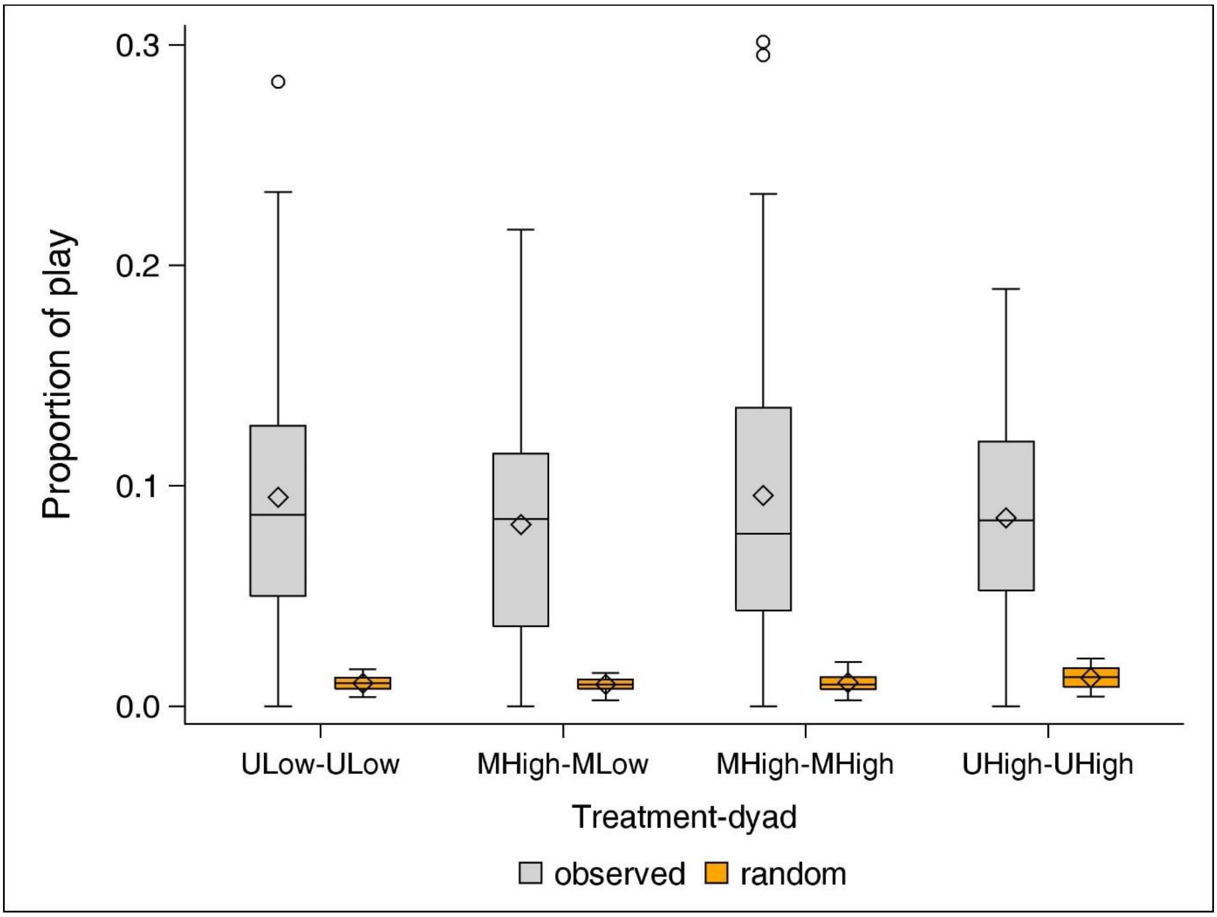

Supplementary Figure S2. Proportion of synchronized dyadic play that was observed as compared to occurring by chance, grouped by treatment-dyad. Diamonds are estimated means.
